# Optically Controlled Terahertz Dynamic Beam Splitter with Adjustable Split Ratio

**DOI:** 10.3390/nano12071169

**Published:** 2022-03-31

**Authors:** Shan Yin, Dehui Zeng, Yuting Chen, Wei Huang, Cheng Zhang, Wentao Zhang, Yiwen E

**Affiliations:** 1 Guangxi Key Laboratory of Optoelectronic Information Processing, School of Optoelectronic Engineering, Guilin University of Electronic Technology, Guilin 541004, China; syin@guet.edu.cn (S.Y.); mailzengdh@163.com (D.Z.); cyt5124@163.com (Y.C.); 2 Hubei Engineering Research Center of RF-Microwave Technology and Application, School of Science, Wuhan University of Technology, Wuhan 430070, China; czhang2020@whut.edu.cn; 3 The Institute of Optics, University of Rochester, Rochester, NY 14627, USA; ye2@ur.rochester.edu

**Keywords:** beam splitter, metasurface, terahertz, dynamic

## Abstract

The beam splitter is an important functional device due to its ability to steer the propagation of electromagnetic waves. The split-ratio-variable splitter is of significance for optical, terahertz and microwave systems. Here, we are the first (to our knowledge) to propose an optically controlled dynamic beam splitter with adjustable split ratio in the terahertz region. Based on the metasurface containing two sets of reversed phase-gradient supercells, we split the terahertz wave into two symmetrical beams. Associated with the reconfigurable pump laser pattern programmed with the spatial light modulator, dynamic modulation of the split ratio varying from 1:1 to 15:1 is achieved. Meanwhile, the beam splitter works at a split angle of 36° for each beam. Additionally, we obtain an exponential relationship between the split ratio and the illumination proportion, which can be used as theoretical guidance for beam splitting with an arbitrary split ratio. Our novel beam splitter shows an outstanding level of performance in terms of the adjustable split ratio and stable split angles and can be used as an advanced method to develop active functional devices applied to terahertz systems and communications.

## 1. Introduction

The manipulation of electromagnetic (EM) waves is significant for optoelectrical devices applied in communications [1], imaging [2] and detection [3]. Metasurfaces, emerging artificial microstructures that can manipulate EM waves, have attracted significant attention. With well-designed structures, metasurfaces can modulate the amplitude [4,5,6], phase [7], polarization [8], and chirality [9]. Owing to their excellent ability of control the wavefront with a phase-gradient configuration, metasurfaces have been developed for various functional devices like the meta-lens [10,11], beam splitter [12,13], and vortex generator [14,15].

The beam splitter is an important component of optical, microwave, and terahertz systems, because it can deflect and divide EM waves to expected angles [16]. Metasurface-based beam splitters were recently reported on. Niu et al. proposed a terahertz reflectarray as a polarizing beam splitter [17]. Ni et al. demonstrated a broadband non-polarizing terahertz beam splitter based on an all-dielectric metasurface [18]. Further, active devices that dynamically manipulate beam splitting have been proposed. The active metasurface terahertz deflector was presented and used to modulate the intensity of the deflected waves via the bias voltage [19]. Graphene-based metasurfaces were shown to dynamically steer the deflection angle by changing the Fermi energy [20]. A coding scheme was introduced to generate more beams by arranging different coding sequences [21,22]. Zhang et al. employed the Pancharatnam–Berry (PB) coding metasurface to form spin-controlled multiple beams with different polarizations [23]. Li et al. presented electronic controlled tunable dielectric metasurfaces modulated by liquid crystal with a beam deflection angle of 11° [24]. Previous work mainly focused on varying the beam splitting number, switching the deflection angles of different beams, or simultaneously modulating the intensity of beams, but active beam splitters with an adjustable split ratio have rarely been reported. In 2020, Kocer et al. presented a dynamic beam splitter employing an elastic substrate based all-dielectric metasurface to undergo splitting in three ways with different split angles and power ratios [25]. However, a dynamic beam splitter that can change the split ratio and maintain the same split angles has not yet been developed.

In this paper, we propose, for the first time (to our knowledge), a photo-induced terahertz beam splitter with an adjustable split ratio at certain split angles. The beam splitter is based on a metasurface composed of metallic structures deposited on the substrate of silicon on sapphire (SOS). The metallic structures consist of two groups of phase-gradient C-shaped split-ring resonators (SRRs). With the inverse phase gradient, the two groups of SRRs diffract the incident waves at two different angles. Combined with the pump laser pattern programmed by digital micromirror devices (DMD), we can control the illuminated area of the metasurface and, therefore, excite photoinduced carriers of the silicon layer. The structures located in the illuminated part will be opaque due to the metallized silicon, which reduces the intensity of the corresponding split beam. Accordingly, the split ratio can be arbitrarily adjusted by choosing the illumination ratio via the patterned pump light. We demonstrate that the split ratio of two beams can dynamically vary from 1:1 to 15:1 with an unchanged deflection angle of 36° for each beam. Implemented by the phase-gradient metasurface combined with the reconfigurable pump laser pattern, our novel beam splitter exhibits an excellent performance regarding the dynamic terahertz beam steering concept, and this optically controlled scheme provides an advanced method to develop more active functional devices that can be applied to terahertz systems and communications.

## 2. Design and Simulation

**Figure 1:**
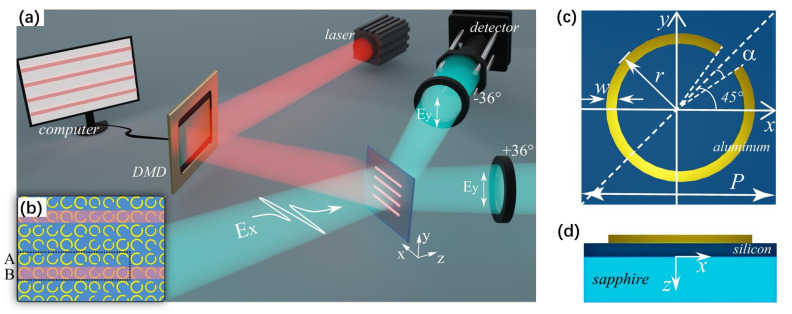
(**a**) Schematic of the proposed beam splitter combined with the optically controlled scheme. (**b**) Partial detailed view of the beam splitter. The dashed frame denotes the basic unit (A + B). (**c**) The geometrical parameters of an SRR. (**d**) Schematic of the multilayered structure.

Our proposed terahertz beam splitter combined with the optically controlled scheme is presented in Figure 1a, and a detailed view of the beam splitter is shown in Figure 1b. C-shaped metallic split-ring resonators (SRRs) are deposited on the top surface, and 200 nm thick aluminum with a conductivity level of 3.56 × 10^7^ S/m is used as a metallic material [26]. The substrate is a 1.5 µm thick silicon layer grown on a 500 µm thick sapphire wafer, and the permittivities of silicon and sapphire are 11.7 and 10.5, respectively [27]. For all simulations, the *x*-polarized terahertz plane wave was normally incident to the metasurface, and the *y*-polarized electric field was detected. The red stripes denote the reconfigurable pump laser pattern illuminating the SRRs and the silicon layer, which can be programmed by the digital micromirror devices (DMD) via a computer.

For each SRR whose structure is presented in Figure 1c,d, the width of the ring is *w* = 5 µm, the angle of the gap is 2*a*, the external radius of the ring is *r*, and the axis of symmetry is rotated by *α* relative to the *x*-axis. The SRRs are aligned in the *x* and *y* directions with a period of *P* = 80 µm. Due to the asymmetry of the SRR under *x*-polarized wave incidence, the polarization can be partially converted to the orthogonal direction in a specific frequency range, and when *α* = ±45°, the polarization conversion ratio is maximized [13]. Therefore, the phase and amplitude of the orthogonally polarized wave can be controlled simultaneously by varying the radius (*r*) and angle of the gap (*a*) of the SRRs.

**Figure 2:**
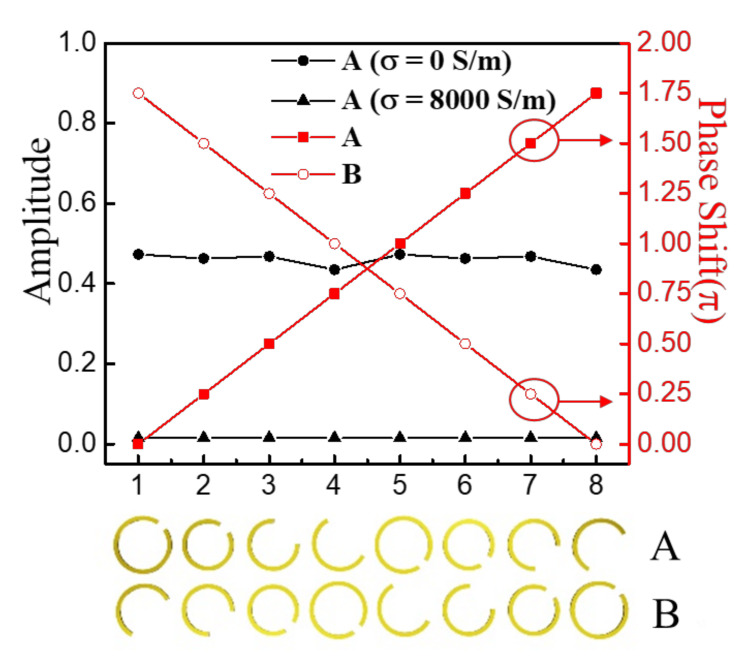
Transmission amplitudes and phases of eight SRRs in supercells A and B at 0.8 THz with the *x*-polarized terahertz incidence and *y*-polarized detection.

In our previous work [11], the amplitude and phase of C-shaped SRRs were investigated. We chose eight pairs of specific parameters (*a* and *r*) of SRRs, as listed in Table 1, and their transmission phases and amplitudes at 0.8 THz are shown in Figure 2. Obviously, the phase of the eight SRRs successively increased by π/4, and the amplitude remained unchanged. Accordingly, we configured the eight SRRs as a positive phase-gradient supercell A whose phase uniformly ranged from 0 to 2π. Additionally, we inversely ordered the eight SRRs to construct another negative phase-gradient supercell B, whose phase uniformly decreased by π/4. Hence, we assembled the two supercells with inverse phase gradients as a basic unit (A + B) of the array, as shown in the inset of Figure 1a. It was possible to generate the two opposite deflected wavefronts, namely two splitting beams, simultaneously. The deflection angle depends on the phase gradient value of metasurfaces, and the relationship between the two obeys the generalized Snell’s law of refraction defined as [7]:

(1) $$n_{t}\sin\theta_{t}-n_{i}\sin\theta_{i}=\frac{\lambda_{0}}{2\pi }\frac{d\Phi }{dx}$$

where $\theta_{t}$ and $\theta_{i}$ represent the refractive and incident angles, respectively; $n_{t}$ and $n_{i}$ are the refractive indexes of the vacuum and sapphire, respectively; $\lambda_{0}$ indicates the wavelength in a vacuum; and dΦ/dx is the phase gradient of the metasurface. In our designed metasurface, the incident angle is $\theta_{i}$ = 0, $n_{t}=1$, and $n_{i}=3.24$. The wavelength $\lambda_{0}$ corresponds to a frequency of 0.8 THz. dΦ/dx=2π/D (*D* = 640 μm is the period of the supercell in *x*-direction). Therefore, by containing the positive and negative phase gradients simultaneously, the deflection angles of the two splitting beams were both calculated as ±35.87° for our beam splitter.

To modulate the intensity of each splitting beam, we simulated the transmission amplitude of the SRRs excited by an external pump laser. The conductivity of photoconductive silicon *σ_s_* is variable with the optical pump power [13,28]. When the metasurface is illuminated by the pump laser, the photoinduced carriers of the silicon layer will be excited, and *σ_s_* = 8000 S/m was used to model the high conductivity of silicon. Due to the increased reflection and absorption of the terahertz wave in silicon under the pump, the transmission amplitude of the terahertz wave dropped to 0.015, as shown in Figure 2. Without the pump, the silicon layer returned to the static state (the conductivity *σ_s_* is 0 S/m), and the transmission amplitude remained around 0.47. Hence, combined with the pump laser pattern programmed by the DMD, we were able to control the illuminated area of the metasurface, as shown in Figure 1a,b with the red reconfigurable stripe pattern, and therefore, we could modify the intensities of the splitting beams.

## 3. Results and Discussion

Since the beam splitter is formed by an array of two supercells, we were able to selectively illuminate the area of the metasurface, and the proportion of each supercell under illumination could be determined. The silicon located in the illuminated part turned opaque and reduced the intensity of the corresponding split beam. As a result, by controlling the illumination pattern, we were able to adjust the intensity of each splitting beam and, therefore, realize the variable split ratio (SR).

**Figure 3:**
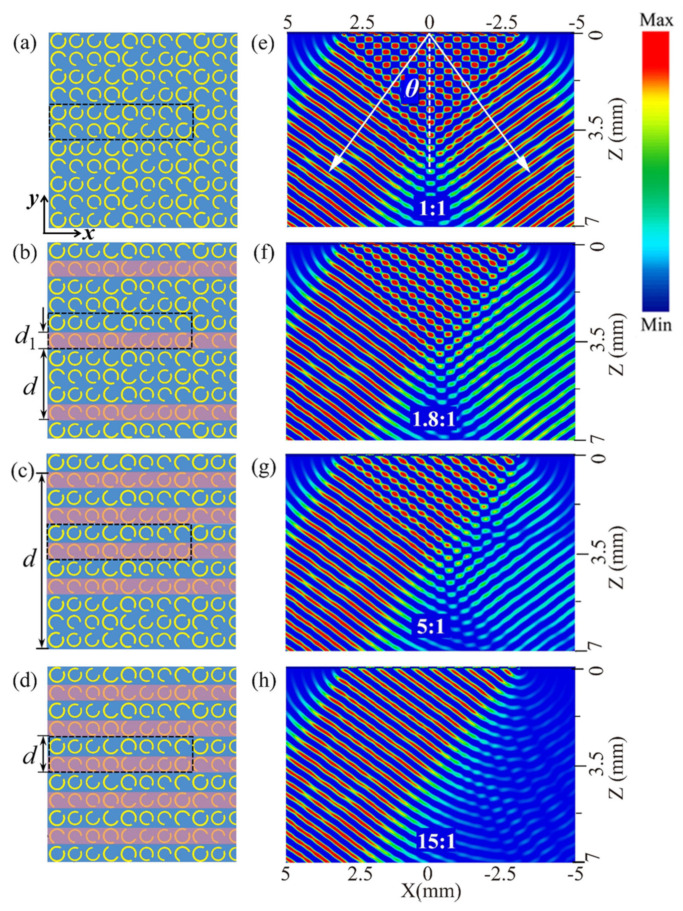
(**a**–**d**) Partial sketches of the designed metasurface with the red stripe patterned illumination. The dashed frames denote the basic unit cells. (**e**–**h**) Simulated electric field distributions of beam splitting in the corresponding illuminated patterns at 0.8 THz.

The function of the beam splitter is displayed in Figure 3. We simulated the electric field distributions of the deflected waves deflected by the beam splitter under four situations with different illuminated patterns at 0.8 THz. Figure 3a shows the situation without a pump laser, and Figure 3e demonstrates the electric field distributions. Beyond the near-field zone of coherent superposition (about 4.1 mm ≈ 10.9λ), two deflected beams were symmetrically split when the terahertz wave was normally incident to the top metasurface. Generally, the split ratio (SR) can be defined as the ratio of the two splitting beams [29]:

(2) $$\mathrm{SR}=\frac{I_{left}}{I_{right}}$$

where *I_left_* and *I_right_* represent the electric field intensities of the outgoing left and right beams, respectively. Here, the left and right beams were deflected by supercells A and B, respectively. Consequently, *I_left_* and *I_right_* were inversely correlated to the illuminated areas of supercells A and B, respectively. In this case, there was no illuminated area, so the intensities of the two splitting beams were equal (the numerical value was about 0.3 V/m), and the SR of our beam splitter was 1:1.

In Figure 3b, the horizontal stripe patterned pump light (denoted with red stripes) is used to illuminate the area of supercell B, where the width of each stripe is *d*_1_ = *P* = 80 µm and is designed to overlap supercell B. With a period of *d* = 4*P* covering 2 basic units, the stripe pattern illuminated half of the supercell B area; hence, the intensity of the right beam attenuated to about half the value of the original intensity (see Figure 3f), while the intensity of the left beam remained at the original level because supercell A was not pumped. As a result, the SR became 1.8:1 (0.30:0.17) in this situation. Similarly, by designing the pump laser pattern well and selectively illuminating fractional supercell B, we were able to obtain different split ratios. In Figure 3c, the period of the stripe illuminating pattern is shown to be *d* = 10*P*, which covers 5 basic units, and every period contains four stripes. Hence, 80% of supercell B was illuminated, and the right beam was evidently weakened, as shown in Figure 3g, so that the SR changed to 5:1 (0.30:0.06). This further increased the proportion of supercell B to 100%, corresponding to the pattern shown in Figure 3d, and the period of the stripe shrunk to *d* = 2*P* while supercell B was fully selectively illuminated. The right beam nearly vanished while the left beam maintained the high intensity, leading to the the highest SR of 15:1 (0.30:0.02), as shown in Figure 3h.

**Figure 4:**
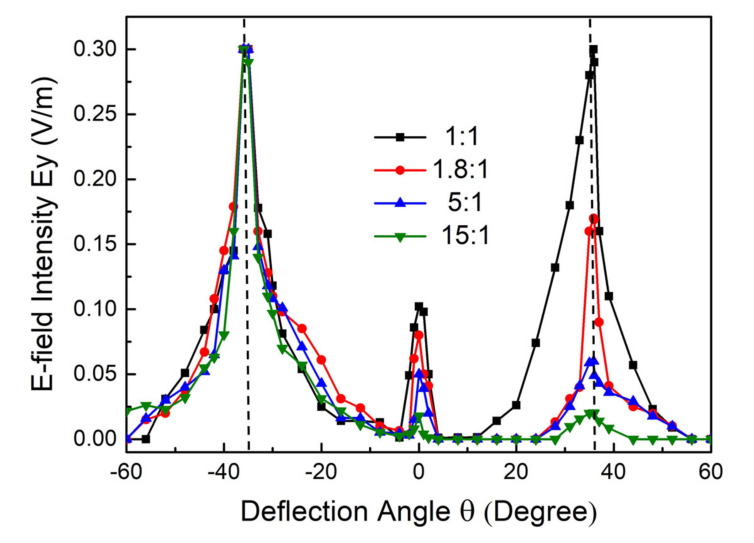
Electric field intensity of reflected waves. The vertical dashed lines denote the maximum values at ±36°.

To clearly present the adjustable property of our beam splitter, we plotted the electric field distributions of the reflected waves versus the deflection angle, as shown in Figure 4. We output the electric field intensity of the splitting beams at a distance of 5 mm on the *xz*-plane. Obviously, the transmitted wave was symmetrically divided in two main directions, and the left split beam maintained the maximal value, while the right one attenuated, corresponding to the cases presented in Figure 3a–d. Note that the deflection angles of the two split beams were consistent in all situations, and the maximum value appeared at ±36° (denoted with the vertical dashed in Figure 4), which is in excellent agreement with Equation (1). Hence, the separation angle between two split beams can reach 72°. Additionally, the split beams were highly concentrated, and the angle of divergence was less than 10°. These results indicate the outstanding performance of our beam splitter in terms of the adjustable split ratio and stable split angles. Compared with previous work [29,30] in which the split ratio or angle were changed by designing different structures, our beam splitter has the advantage of having an active metasurface that can realize multifunctionalization with one sample. This is beneficial for the development of integrated devices in terahertz systems.

**Figure 5:**
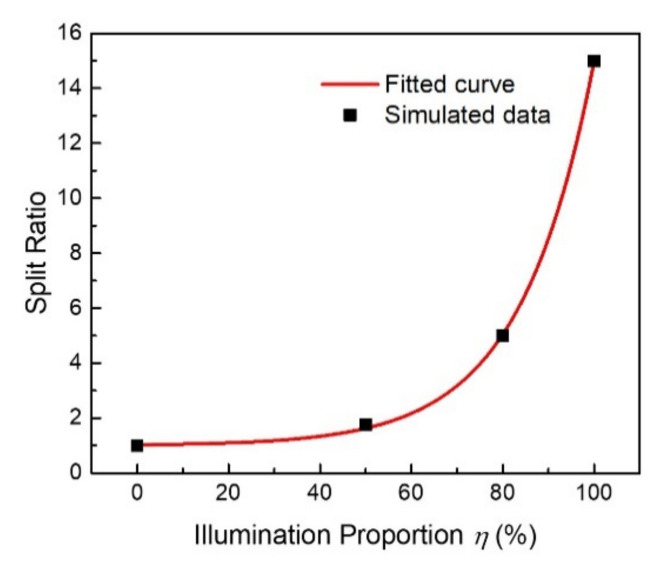
Relationship between the beam split ratio and the illumination proportion of supercell B.

Additionally, we extracted the relationship between the split ratio (SR) and the proportion of the pump illumination area for supercell B (*η*), and the results are plotted in Figure 5 with scattered dots. It was concluded that the split ratio increased with the enlargement of the illumination area, showing an exponentially increasing trend. Since the starting SR was 1:1, the relationship was fitted with the exponential formula *SR* = 1 + 0.028 exp (0.062*η*). As shown in Figure 5, the fitted curve (red line) agrees well with the simulated data (dots). Based on the *SR*-*η* formula, theoretically, the arbitrary intensity of the split beam can be manipulated through the optically controlled system by editing the pump pattern via DMD in real-time. For experimental feasibility, reconfigurable terahertz grating was demonstrated with a silicon wafer using DMD [31], and it was shown that the spatial profile at the silicon wafer can be controlled at a resolution of 10 μm [32], so it is likely that the flexible modulation of the split ratio in our beam splitter could be achieved through association with the reconfigurable pump laser pattern. Until now, this optically controlled scheme has not been applied to the dynamic beam splitter, and our proposal can provide a flexible means to manipulate the terahertz wave.

## 4. Conclusions

A novel beam splitter with an arbitrary controllable split ratio in the terahertz region was proposed. Based on the metasurface containing two sets of reversed phase-gradient supercells, we split the terahertz wave into two symmetrical beams. Through an association with the reconfigurable pump laser pattern programmed with the spatial light modulation scheme, dynamic modulation of the split ratio varying from 1:1 to 15:1 was achieved. Meanwhile, the beam splitter was shown to work at a deflection angle of 36° for each beam, and the separation angle between two split beams could exceed 70°. We obtained an exponential relationship between the split ratio and the illumination proportion, which could be used as theoretical guidance for beam splitting with an arbitrary split ratio. To the best of our knowledge, this is the first proposal of a terahertz beam splitter with a dynamically controllable split ratio at stable split angles. We believe that this design represents an advanced way to develop more active function devices and to promote their practical application in optical, microwave, and terahertz systems.

## 5. Patent

Shan Yin, Dehui Zeng, Wei Huang, Ling Guo, Wentao Zhang, Xianming Xiong. A dynamic adjustable terahertz beam splitter based on composite metasurface. 202210034731.2 (China).

## Figures and Tables

**Table 1:** Geometrical parameters of the eight SRRs forming the supercell A.

Number	1	2	3	4	5	6	7	8
*r* (μm)	35	31.5	32	34.55	35	31.5	32	34.55
*a* (°)	10.5	15	43	73.5	−10.5	−15	−43	−73.5

## Data Availability

The raw data are available upon request from the authors.
